# Supplementary material for: Transcriptomic Insights into Mechanisms of Early Seed Maturation in the Garden Pea (Pisum sativum L.)
Source: Cells. 2020 Mar 23;9(3):779. doi: 10.3390/cells9030779 (PMC7140803; doi:10.3390/cells9030779)

# Zhongwan−6,25(1)

## Global Summary

%DE = 0.24

# genes with fdr < 0.2 = 6303 ( 2858 + / 3445 −)

# genes with fdr < 0.1 = 5917 ( 2679 + / 3238 −)

# genes with fdr < 0.05 = 5467 ( 2469 + / 2998 −)

# genes with fdr < 0.01 = 4736 ( 2155 + / 2581 −)

# genes in genesets = 0

<FC> = 0

<t−score> = 0

<p−value> = 0

<fdr> = 0.76

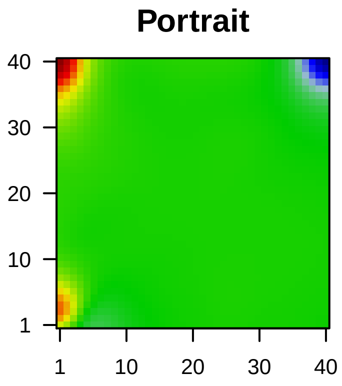


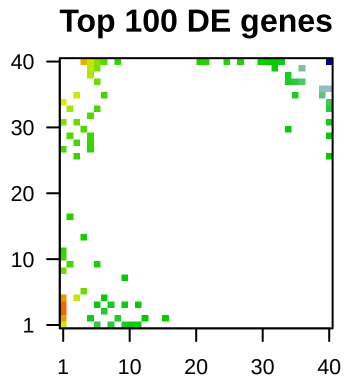


## Global Genelist

| Rank | ID | log(FC) | p−value | fdr | Metagene | Description |
|---|---|---|---|---|---|---|
| 1 | TRINITY_DN2 | −0.06 | [illegible] | [illegible] | [illegible] | |
| 2 | TRINITY_DN1 | 0.06 | [illegible] | [illegible] | [illegible] | |
| 3 | TRINITY_DN4 | 0.07 | [illegible] | [illegible] | [illegible] | |
| 4 | TRINITY_DN4 | 0.04 | [illegible] | [illegible] | [illegible] | |
| 5 | TRINITY_DN4 | −0.15 | [illegible] | [illegible] | [illegible] | |
| 6 | TRINITY_DN3 | −0.03 | [illegible] | [illegible] | [illegible] | |
| 7 | TRINITY_DN3 | −0.03 | [illegible] | [illegible] | [illegible] | |
| 8 | TRINITY_DN3 | −0.04 | [illegible] | [illegible] | [illegible] | |
| 9 | TRINITY_DN3 | 0.04 | [illegible] | [illegible] | [illegible] | |
| 10 | TRINITY_DN3 | 0.05 | [illegible] | [illegible] | [illegible] | |
| 11 | TRINITY_DN3 | 0.06 | [illegible] | [illegible] | [illegible] | |
| 12 | TRINITY_DN1 | −0.1 | [illegible] | [illegible] | [illegible] | |
| 13 | TRINITY_DN1 | −0.14 | [illegible] | [illegible] | [illegible] | |
| 14 | TRINITY_DN1 | 0.04 | [illegible] | [illegible] | [illegible] | |
| 15 | TRINITY_DN1 | −0.12 | [illegible] | [illegible] | [illegible] | |
| 16 | TRINITY_DN1 | 0.09 | [illegible] | [illegible] | [illegible] | |
| 17 | TRINITY_DN5 | −0.04 | [illegible] | [illegible] | [illegible] | |
| 18 | TRINITY_DN1 | 0.1 | [illegible] | [illegible] | [illegible] | |
| 19 | TRINITY_DN1 | 0.08 | [illegible] | [illegible] | [illegible] | |
| 20 | TRINITY_DN2 | −0.04 | [illegible] | [illegible] | [illegible] | |

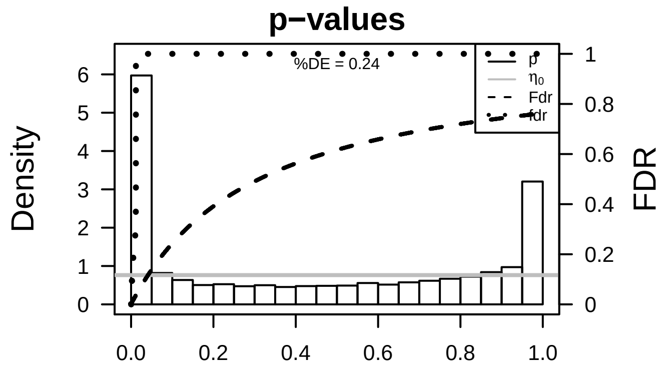

Supplement: Supplementary file 1 [file cells-09-00779-s001.zip › Supplementary materials/FileS2.zip!/FileS1/Summary Sheets - Samples/Zhongwan.6.25.1..pdf]
